# Antiquorum and Antibiofilm Activities of *Piper bogotense* C. DC. against *Pseudomonas aeruginosa* and Identification of Bioactive Compounds

**DOI:** 10.3390/plants12091901

**Published:** 2023-05-06

**Authors:** Andrés G. Sierra-Quitian, Lida V. Hernandez-Moreno, Ludy C. Pabon-Baquero, Juliet A. Prieto-Rodriguez, Oscar J. Patiño-Ladino

**Affiliations:** 1 Departamento de Química, Facultad de Ciencias, Universidad Nacional de Colombia, Sede Bogotá, Bogotá 111321, Colombia; asierraq@unal.edu.co (A.G.S.-Q.); lhernandezmo@unal.edu.co (L.V.H.-M.); ojpatinol@unal.edu.co (O.J.P.-L.); 2 Escuela de Ciencias Básicas y Aplicadas, Universidad de La Salle, Bogotá 111711, Colombia; lupabon@unisalle.edu.co; 3 Departamento de Química, Facultad de Ciencias, Pontificia Universidad Javeriana, Bogotá 110231, Colombia

**Keywords:** *Piper bogotense*, piperaceae, *Pseudomonas aeruginosa*, virulence factors, biofilm formation, quorum sensing

## Abstract

The present study describes the anti-biofilm and quorum sensing (QS) inhibitory potential of extracts and chemical constituents from *Piper bogotense*. Antibiofilm potential was determined through crystal violet assay against *Pseudomonas aeruginosa*, while QS inhibition efficacy was determined through violacein inhibition assay using *Chromobacterium violaceum* as a bacterial model. Additionally, this study reports the effects of the chemical constituents isolated in *P. bogotense* against various virulent factors associated with QS, such as the percentage decrease in pyocyanin, elastase, and protease production. The chemical study led to the isolation and identification of two prenylated benzoic acids (**1** and **2**) and a prenylated hydroquinone **3**, of which compounds **1** and **2** are reported for the first time for *P. bogotense*. The ethanolic extract and the DCM fraction from *P. bogotense* stand out for reducing violacein production in *C. violaceum*, as well as the biofilm formation in *P. aeruginosa*. Compounds **2** and **3** stand out for having the lowest violacein production (43.8% and 68.3%), as well as the lowest production of virulence factors such as elastase (60.2% and 51.4%) and pyocyanin (39.7% and 33.2%). These results demonstrate the potential of *P. bogotense* components to be used as an alternative control against multidrug-resistant *P. aeruginosa*.

## 1. Introduction

Antimicrobial resistance (AMR) is one of the top 10 global public health threats due to the increased burden of infectious diseases and high costs of medical care; it is estimated that AMR can cause around 10 million deaths a year by 2050 [1,2]. Among the control alternatives to reduce AMR is the inhibition of the quorum sensing system (QS), a cellular communication mechanism that controls virulence factors and the production of biofilms, which favors the survival of bacteria in the host [3,4]. The importance of controlling this system lies in controlling the virulence factors associated with QS, reducing the evolutionary pressure generated in bacteria when treated with antibiotics, and prolonging their lifespan using combination therapies [5,6,7]. *Pseudomonas aeruginosa* is a Gram-negative pathogen of clinical importance since it is one of the most common agents of infections observed in Intensive Care Units (ICU) and is responsible for high mortality. [8,9]. It is reported as one of the main microorganisms causing nosocomial infections in developed countries, with a prevalence of 3–12%, and in underdeveloped countries, between 5–19% with a predisposition to be multidrug-resistant [10,11,12,13,14]. This bacterium can form biofilms and produce virulence factors that help to impede the action of antibiotics and promote their pathogenicity in the host, which is the reason it has been declared by the World Health Organization (WHO, Geneva, Switzerland) as a critical priority pathogen as it presents multidrug resistance to a wide range of antibiotics [15,16,17,18,19]. QS controls the virulence factors and the formation of biofilms, so the search for anti-QS strategies could be a potential objective to prevent infection by *P. aeruginosa*.

Plants have been considered a source of various bioactive substances, including some with antibacterial and anti-QS potential. Regulation of biofilm formation, Inhibition of QS, and attenuation of virulence factors in *P. aeruginosa*, have been reported for a wide variety of extracts, essential oils, and chemical constituents [20,21,22,23,24,25]. Plant-derived secondary metabolites with the potential to inhibit QS include phenols, flavonoids, coumarins, terpenoids, amides, phenylpropanoids, alkaloids, and benzoic acid derivatives, among others [23,24,25]. Although many herbal extracts exhibit anti-QS effects, investigations are needed to determine some bioactive species’ chemical constituents and characterize the molecular mechanisms involved in the control and perturbation of QS signaling.

Among the plant families where some of its species have shown potential for QS control is the Piperaceae family, which is recognized worldwide for its economic importance due to its applications in food, industry, and medicine [26,27,28]. The *Piper* genus is one of the most representative of the family and comprises about 1457 species distributed worldwide, mainly in tropical and subtropical regions [29]. Many chemical constituents have been found in species of the *Piper* genus, highlighting the presence of amides, alkylbenzenoids, flavonoids, terpenoids, phenylpropanoids, and prenylated benzoic acid derivatives [30,31]. The antimicrobial potential of species of this genus against different pathogenic bacteria has been described in the literature, including *P. aeruginosa* [32,33,34,35]. However, there are few studies aimed at determining the effect of products derived from species of *Piper* genus to inhibit QS and regulate the biofilm formation of *P. aeruginosa* [36,37,38,39,40,41,42]. The antiQS on *C. violaceum* and antibiofilm on *P. aeruginosa* potential of extracts from *P. betle*, *P. nigrum* and *P. longum* have been reported in the literature [37,38,40,41]. The ability to inhibit the production of pyocyanin and the swarming motility of *P. aeruginosa* was reported for the extract from *P. betle* [37,41]. The EOs from *P. bogotense*, *P. brachypodom* and *P. bredemeyeri*, showed inhibiting QS on *C. violaceum* CV026 with IC_50_ of 514, 93 and 45 μg/mL, respectively [43]. The essential oil of *P. nigrum* at 50 μL/mL caused a 26% inhibition in the formation of *P. aeruginosa* biofilms and a 16% inhibition in the production of violacein [41,44]. Investigations on two amides (piperine and trichostaquine) present in the extract of *P. nigrum* showed that they have the potential to inhibit the production of violacein from *C. violaceum*, while they cannot inhibit the biofilm formation of *P. aeruginosa* [40]. Flavonoids from *P. delineatum* exhibited the ability to disrupt QS-mediated bioluminescence downstream LuxO and showed a strong dose-dependent inhibition of biofilm formation in the bacterial model *Vibrio harveyi* [39].

*Piper bogotense* C. DC. (Synonyms: *P. barbatum* C. DC, *P. duriraneum* C. DC, *P. fistulosum* C. DC), popularly known as matico, cordoncillo, bordoncillo, or pipilongo, is a native species distributed in tropical countries such as Ecuador, Colombia and Panama [28,45]. This species is of great agroforestry interest in Colombia due to its medicinal applications, uses as fodder and biofertilizer, and because it contributes to the protection and recovery of soils [46,47]. Additionally, the ripe fruits of *P. bogotense* are a source of food for bats and native and migratory birds [47]. In traditional medicine, this species is used as a healing, anti-inflammatory, analgesic and for the treatment of hepatic pain [48,49]. The presence of prenylated hydroquinones and benzoquinones from fruits, as well as flavonoids, alkaloids, lactams and sterols from stems and leaves, have been reported in *P. bogotense* [43,50,51,52,53]. The essential oil from its leaves mainly contains *trans*-sabinene hydrate, α-phellandrene, α-pinene and limonene [43,53,54]. In addition to the previously mentioned antiQS potential for this essential oil, its antifungal and antiparasitic activity has been determined [53,55]. Cytotoxic activity against various cell lines has been reported for extracts from *P. bogotense* [56,57].

The present investigation describes the anti-QS activity on *C. violaceum*, and antibiofilm and antivirulence effects on *P. aeruginosa* of extracts and chemical compounds from *P. bogotense*.

## 2. Results and Discussion

### 2.1. Phytochemical Study and Anti-QS and Anti-Biofilm Activity

The phytochemical study of the aerial part of *P. bogotense* led to the obtaining of five fractions of different polarity (DCM, EtOAc, IPA, EtOH:H_2_O). The fractions and the extract did not show significant effects (*p* < 0.05) on the growth of *P. aeruginosa* at the concentrations evaluated, while positive control gentamicin affected total growth inhibition at 2 µg/mL (Table S1 in Supplementary Material). Previous reports of *Piper* species that have been evaluated against *P. aeruginosa* show that some do not affect bacterial growth, as reported for *P. betle* at concentrations of 150–50 ppm and *P. nigrum* at 3.2 and 1 mg/mL [33,37]. However, other *Piper* species have been shown to affect *P. aeruginosa* growth at higher concentrations than those evaluated in this study. For example, the ethanolic extract of *P. tuberculatum* a MIC > 1024 µg/mL is reported [58], while *P. longum* and *P. nigrum* are reported inhibition halos higher than 18 mm at concentrations between 5–10 mg/mL in the disk diffusion assay [34]. In the case of *P. umbelatum*, DCM extract has been reported to have the potential to inhibit the growth of *P. aeruginosa* ATCC 27853 at 1000 µg/mL [35]. The results of this assay allowed us to determine that the concentrations evaluated did not affect the growth of *P. aeruginosa* to avoid selective pressure and prevent resistance; therefore, these were selected for subsequent assays.

**Figure 1:**
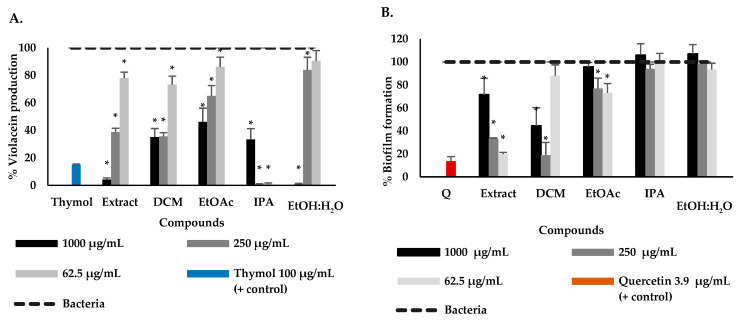
Effect of the extract and fractions of *P. bogotense* on the production of violacein of *C. violaceum* (**A**) and the formation of biofilm in *P. aeruginosa* (**B**). Data are represented by the mean ± standard deviation of five independent replicates. * Indicate a significant difference according to Duncan’s test (*p* < 0.05).

Subsequently, *P. bogotense* extract and fractions were evaluated on the attenuation of *C. violaceum* QS in the violacein production assay. The results showed that the extract and DCM, EtOAc and EtOH:H_2_O fractions reduced violacein production directly proportional to the concentration, contrary to the IPA fraction, where an inverse relationship was observed. It was found that the extract and IPA and EtOH:H_2_O fractions had a violacein production lower than 5% at the highest concentration, being even lower than the inhibition control thymol (Figure 1A). Regarding studies of the anti-QS activity of the ethanolic extract of *P. bogotense* this is the first report; however, previously, it has been reported that the essential oil of this species, whose major components correspond to *trans*-sabinene, α-pinene, and α-phellandrene, has violacein inhibitory effects on *C. violaceum* strain CV026 with IC_50_ of 513.8 μg/mL [43]. Previous studies found that the extracts of *P. betle* and *P. nigrum* showed anti-QS activity in the quantification assay of violacein produced by *C. violaceum* using concentrations between 1 and 3 mg/mL [37,42].

Next, the ability to reduce the formation of *P. aeruginosa* biofilms by exposure to the extract and fractions of *P. bogotense* is evaluated. Figure 1B shows the results, finding that the extract and the DCM fraction were the only ones that managed to reduce biofilm formation significantly. For the ethanolic extract, an inversely proportional effect to the concentration was found. It was evidenced that the extract significantly reduced biofilm formation with a percentage of formation of 19% at 62.5 μg/mL, followed by the DCM fraction, which had a percentage of formation of 18.9% at 250 μg/mL being these results close to the effect of the quercetin inhibition control which produced 13% at 3.9 μg/mL. For the EtOAc fraction, a slight reduction in biofilm formation was observed, with a percentage of formation of approximately 70% at the lowest concentrations. For this species, the effect on *P. aeruginosa* biofilm formation has not been reported; however, for other species of the genus, it has. For example, for *P. betle* it is found that the ethanolic extract of leaves inhibits *P. aeruginosa* PAO1 biofilm by 75% at 200 μg/mL [37,41], and for *P. nigrum,* it is reported for the ethanolic extract of seeds that it does not affect *P. aeruginosa* biofilm at 500 μg/mL [40]. Considering the results obtained, the DCM fraction was chosen for the purification and identification of the bioactive compounds because it inhibited the production of violacein from *C. violaceum* and because it was the fraction that had the best effect on the reduction of the *P. aeruginosa* biofilm.

**Figure 2:**
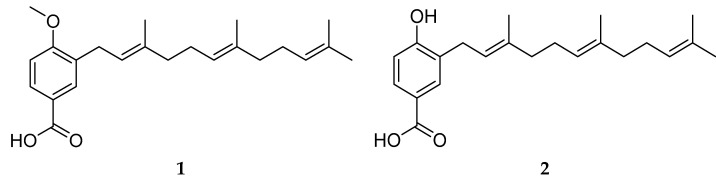

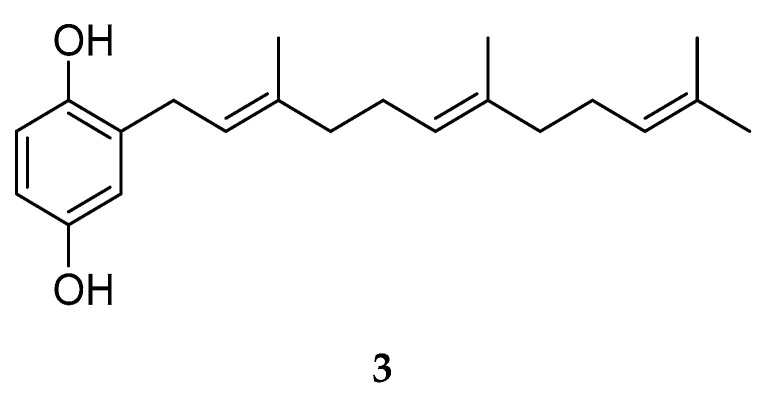
Chemical constituents isolated and identified from *P. bogotense*.

The phytochemical study carried out on the DCM fraction from the aerial part of *P. bogotense* led to the isolation of three compounds, which were identified by spectroscopic methods and by comparison with data reported in the literature. In this way, two derivatives of benzoic acid (**1** and **2**) and hydroquinone (**3**) were isolated and identified (Figure 2). This is the first report of **1** and **2** for the species, while **3** has been previously described in fruits from *P. bogotense* [52]. All compounds have been identified in other species of the genus *Piper* and their structural characteristics agree with the chemotaxonomy of the genus. In some of its species, it is common to observe the presence of benzenoids with carboxyl, oxygenated and/or prenylated substituents [31].

NMR analysis for compound **1** (see Figures S1 and S2 in Supplementary Material ) indicated the presence of a 1,3,4-trisubstituted aromatic ring by the signals in ^1^H-NMR at δ_H_ 7.99 (d, *J* = 8.6 Hz, 2.2 Hz, 1H, H-2), 7.91 (s, *J* = 2.2 Hz, 1H, H-6) and 6.88 (d, *J* = 8.6 Hz, 1H, H-3) and signals in APT with δ_C_ 161.7 (C), 131.3 (CH), 109.4 (CH), 130.1 (CH), 121.2 (C), and 130.0 (C). Additionally, NMR analysis determined the presence of a carboxyl group, a methoxy group, and a farnesyl-type aliphatic chain located on the aromatic ring, which by comparison with literature data, allowed the identification of **1** as 3-farnesyl-4-methoxybenzoic acid [59]. This compound has been previously described in *P. marginatum* [31,59,60], however, its biological properties are unknown.

Compound **2** presented an NMR profile like **1**. The difference between their spectra was because for **2** the characteristic signal of the methoxy group was not observed in ^1^H-NMR. However, in APT at δ_C_ 158.0, a characteristic signal of oxygenated aromatic carbon was observed, which suggested the presence of a hydroxyl group in position 4 of the aromatic ring. From the analysis carried out and by comparison with the data reported in the literature, **2** was identified as 3-farnesyl-4-hydroxybenzoic acid [61], which has been previously reported in *P. auritum*, *P. marginatum*, *P*. *obliquum*, *P. tricuspe* and *Dictyopteris divaricate* [61,62,63,64]. This compound has been described in the literature for its antiparasitic (*Plasmodium falciparum*), antibacterial (*Escherichia coli* and *Bacillus subtilis*) properties and cell lines (MCF-7, H-460, and SF-268) [57,62], and for its ability to inhibit *β*-secretase-1, an important enzyme in the development of Alzheimer’s disease [36].

Although compound **3** presents an NMR profile like **1**, it does not present characteristic signals for the carboxyl and methoxy groups. Additionally, the spectroscopic analysis led to the determination that **3** has a farnesyl-type substituent and two hydroxyl groups located on the aromatic ring. By comparison with the spectroscopic data reported in the literature, **3** was identified as 2-farnesylhydroquinone [52], which has been previously reported from *P. bogotense* and *P. tricuspe* [52,62]. The antiparasitic activity against *P. falciparum* was described for **3** with an IC_50_ of 1.37 μM [62].

### 2.2. Effect of P. bogotense Compounds against C. violaceum Quorum Sensing, Biofilm Formation and Production of Virulence Factors in P. aeruginosa

In the bacterial growth assay, it was observed that the different evaluated concentrations of the compounds (250–62.5 μg/mL) had no significant effect on the growth of *P. aeruginosa* compared to the control, with a statistical significance level of *p* < 0.05 (Table S4 in Supplementary Material). These results allowed us to identify that the evaluated concentrations did not affect the growth of bacteria and were chosen for further testing; thus, resistance could be decreased by not causing selective pressure [65]. This is the first report on the effect of compounds **1** to **3** on *P. aeruginosa*. In previous studies, it has been reported that prenylated derivatives of benzoic acid and prenylated hydroquinones have inhibitory effects on the growth of *P. aeruginosa*. For example, 3-farnesyl-2-hydroxybenzoic acid has a MIC of 6.25 μg/mL [64,66], and 2-farnesyl-6-methylbenzoquinone has caused mild inhibition of *P. aeruginosa* with 60 µg in disk inhibition assay [67].

**Figure 3:**
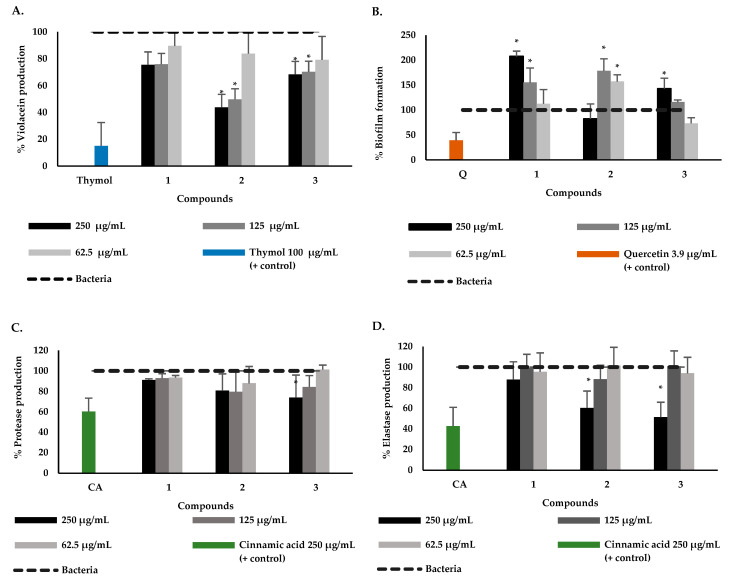

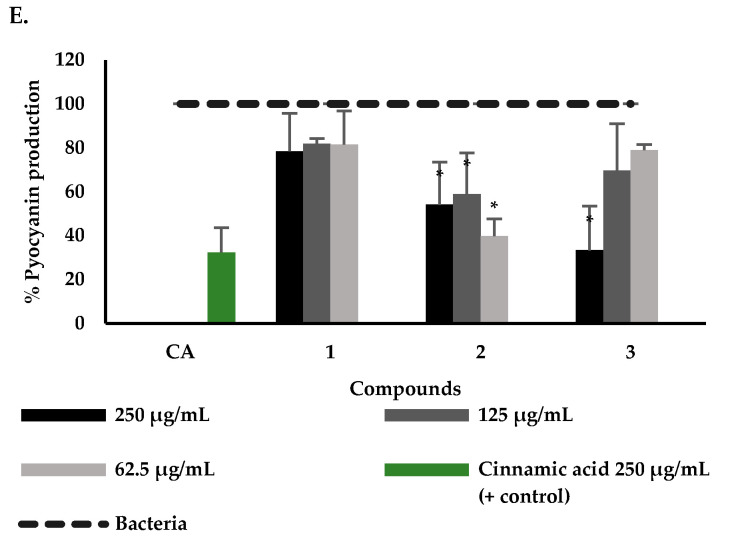
Effect of *P. bogotense* compounds on violacein production in *C. violaceum* (**A**), biofilm formation (**B**), protease (**C**), elastase (**D**), and pyocyanin (**E**) production of *P. aeruginosa*. Data are represented the mean ± standard deviation of five independent replicates. * Indicate a significant difference according to Duncan’s test (*p* < 0.05).

To establish whether compounds **1** to **3** are some of those responsible for the bioactivity observed for the DCM fraction, their effect on violacein production in *C. violaceum* and biofilm formation in *P. aeruginosa* was determined and the results are presented in Figure 3A,B. The results showed that the isolated compounds from *P. bogotense* showed lower activity in inhibiting the production of violacein compared to the extract and DCM fraction. However, it is noteworthy that compounds **2** and **3** significantly reduced the violacein production at the two highest concentrations, with reductions of 43.8% and 49.7% for **2** and 68.3% and 70.2% for **3**, respectively (Figure 3A). These results indicate that compounds of *P. bogotense* can inhibit the QS of the *C. violaceum* strain since violacein is a product of this system. It is worth noting that the quantification of violacein in *C. violaceum* has long been utilized as a biosensor assay to search for QS inhibitors, given that this strain can produce and detect acyl-homoserine lactones (AHL) by itself through the expression of this pigment [68,69,70,71,72].

The study demonstrated that compounds **1** to **3** promoted biofilm formation in *P. aeruginosa*, contrary to what was observed with quercetin (Figure 3B), a compound for which the ability to inhibit *P. aeruginosa* biofilm has previously been reported [73,74]. These compounds have no previous reports on antibiofilm activity, however, other similar compounds, such as benzoic acid derivatives have shown the potential to reduce the biofilm of *P. aeruginosa*. For example, gallic acid has been shown to have the ability to reduce the formation by 30.1% (200 µg/mL) [75] and vanillic acid by 46% (4 mmol/L) [76]. It has also been found that hydroquinone-like compounds, such as avarol and sesquiterpene hydroquinone, exhibited inhibitory effects on *P. aeruginosa* PAO1 biofilm formation at subinhibitory concentrations [77]. It has been reported that phenolic compounds present antimicrobial activity and protect plants from phytopathogenic bacteria. However, in this study, it was found that the evaluated compounds promoted the formation of biofilm in *P. aeruginosa*, an effect that has previously been reported as a strategy that facilitates the survival and protection of the bacteria against this type of compounds [75]. Among the possible mechanisms that explain the increase in the formation of the film is the effect on the synthesis of AHL, finding that the production of 3-oxo-C12-HSL increases under the action of different phenols at subinhibitory concentrations, leading to an effect in the LasI/LasR complex. Additionally, it has been reported that this type of compound does not directly affect the expression of *lasI* and *rhlI* genes, so it is possible that they do not mimic AHL [75].

The results obtained indicate that the compounds have the capacity to affect the QS in *C. violaceum* but not the formation of biofilms in *P. areuginosa*. Taking into account that biofilm formation is a multifactorial process that involves QS, exopolysaccharide, and c-di-GMP systems [77], it cannot be definitively concluded that *P. bogotense* compounds do not act on one of the QS pathways of *P. aeruginosa*. Therefore, to corroborate the effect of these compounds on the QS system of *P. aeruginosa*, their effect on three virulence factors was evaluated. The effect of compounds **1** to **3** on the production of elastases, proteases, and pyocyanin are shown in Figure 3C–E. It was found that only **3** reduced the production of proteases at the highest concentration (250 µg/mL). In the other two factors’ production, it was found that **2** and **3** exerted an inhibitory effect, with a percentage of elastase production of 60% and 51% at (250 µg/mL), respectively, while pyocyanin production was 39% (62.5 µg/mL) and 33% (250 µg/mL), respectively. In previous studies, cinnamic acid was used as an inhibition control and showed inhibitory activity similar to that found in this study on virulence factors at a concentration of 250 µg/mL [78]. This is the first report of activity on *P. aeruginosa* virulence factors of *P. bogotense* compounds. It has been found for similar compounds, such as benzoic acid derivatives, to affect virulence factors; for example, for vanillic acid, its potential to reduce *P. aeruginosa* pyocyanin production by 16% (4 mmol/L) is reported [76] and in other bacteria such as *Serratia marcescens* it inhibits protease biosynthesis by 50% [79]. For other hydroquinone-type compounds, such as avarol, it was reported to reduce *P. aeruginosa* pyocyanin production by 39% [77]. From the analysis of the results obtained with compounds **1** to **3** and the structural characteristics, the following qualitative approximations of structure-activity can be made. When comparing the results obtained with compounds **1** and **2** in the five bioassays, it is observed that compound **1** had no activity in any assay; therefore, it can be indicated that the inactivity of this compound is related to the presence of the methoxyl group in position 4 of the aromatic ring. Comparing now the results found for **2** and **3**, it is possible to emphasize that the presence of the carboxyl group in position 1 of the aromatic ring, instead of a hydroxyl group, favors the inhibitory effect on the production of violacein, elastase, pyocyanin, and proteases.

Considering the results, the capacity of the *P. bogotense* compounds evaluated stands out, since they had an effect on the inhibition of some virulence factors and are considered as an alternative to reduce the pathogenicity of *P. aeruginosa* without affecting growth. It is important to highlight that compounds capable of altering bacterial QS could potentially be used in combination with conventional antibiotics to increase the efficiency of current antimicrobials by reducing the minimum inhibitory concentration, an approach that may also help to overcome antimicrobial resistance and side effects of drugs [76]. The results of this study do not allow us to know the mechanism of action against the QS system of *P. aeruginosa*, so it is important to expand the studies to determine it among the possible mechanisms that have been reported for natural molecules as QS inhibitors may be antagonists, inhibitors of AHL biosynthesis and degradation, and AHL receptor mimetics. This could suggest that these molecules would act on the signals produced by the LasR, RhlR, or PQS system, which regulate the virulence factors evaluated [80,81,82].

## 3. Materials and Methods

### 3.1. General Experimental Procedures

The solvents were technical grade and distilled before use. Thin-layer chromatography (TLC) was performed on SiliaPlate^TM^ alumina plates pre-coated with silica gel 60 F_254_ (SiliCycle^®^ Inc., Quebec, QC, Canada). Vacuum Liquid Chromatography (VLC) was performed on SiliaPlate^TM^ silica gel F_254_ of size 5–20 μm (SiliCycle^®^ Inc., Quebec, QC, Canada). Flash Chromatography (FC) was performed on SiliaFlash^®^ silica gel P_60_ of size 40–63 μm (SiliCycle^®^ Inc., Quebec, QC, Canada). NMR measurements were performed on Bruker Advance AC-400 spectrometer (Bruker^®^, Leipzig, Germany) ^1^H-NMR and APT experiments, operating at 400 MHz for ^1^H and 100 MHz for APT. The reference strains selected in this study were *P. aeruginosa* ATCC BAA-47 and *C. violaceum* ATCC 12472, obtained commercially from the American Type Culture Collection. Azocasein (Sigma-Aldrich^®^, E.E.U.U., Burlington, MA, USA) was used as substrate for the protease assay, and congo red elastin (Sigma-Aldrich^®^, E.E.U.U., Burlington, MA, USA) was used for the elastase assay. An orbital shaker with Jeio tech temperature (Jeio tech^®^, Daejeon, Republic of Korea) was used for the growth of the microorganisms. Optical Density (OD) measurements were performed on a Multiskan sky microplate reader (Multiskan sky Thermo scientific^®^, E.E.U.U., Waltham, MA, USA).

### 3.2. Plant Material

The aerial part of the plant was collected in the “Enrique Olaya Herrera” National Park, located in the city of Bogotá D.C. (Colombia). The species was determined by the biologist Ricardo Callejas, and a specimen of *P. bogotense* C. DC. was deposited in the Universidad de Antioquia Herbarium.

### 3.3. Extraction and Isolation of Compounds

The dried and ground aerial part of *P. bogotense* (1000 g) was subjected to extraction with 96% ethanol using the maceration method at room temperature. The resulting solution was concentrated by distillation under reduced pressure to obtain 458 g of ethanolic extract. A part of the extract (150 g) was fractionated by vacuum liquid chromatography (VLC) using solvents of different polarity: dichloromethane (DCM), ethyl acetate (EtOAc), Isopropanol (IPA) and ethanol–water mixture (EtOH:H_2_O) (80:20). After evaporation of the solvents under reduced pressure, the fractions DCM (40.4 g), EtOAc (20.5 g), IPA (14.0 g) and EtOH:H_2_O (15.0 g) were obtained. The DCM fraction (40.4 g) was subjected to flash chromatography (FC) eluting with a hexane:EtOAc mixture in increasing polarity (95:5 to 50:50), obtaining 75 fractions that were combined into 4 final fractions according to the TLC profile. Fraction 2 (15.6 g) was subjected to purification by repeated FC eluting with a mixture of hexane:EtOAc (97:3), hexane:acetone (80:20), and hexane:EtOAc (70:30), obtaining a crystalline white solid corresponding to 3-farnesyl-4-methoxybenzoic acid **1** (500.2 mg, m.p. 57–59 °C). Fraction 3 (3.6 g) was subjected to successive FC with a mixture of hexane: EtOAc (95:5) and DCM:EtOAc (80:20), obtaining a green solid corresponding to 3-farnesyl-4-hydroxybenzoic acid **2** (70.5 mg, m.p. 52–54 °C). Fraction 4 (1.8 g) was repeatedly fractionated by successive FC with a mixture of DCM:EtOAc:acetone (60:30:10), Hexane:MEK (80:20), hexane:CHCl_3_ (60:40), and hexane:EtOAc (70:30), obtaining a white solid corresponding to 2-farnesyl-1,4-hydroquinone **3** (18.0 mg, m.p. 52–54 °C).

3-farnesyl-4-methoxybenzoic acid (**1**): White solid, melting point (m.p.): 57–59 °C. ^1^H NMR (400 MHz, CDCl_3_): δ_H_ 7.99 (d, *J* = 8.6 Hz, 2.2 Hz, 1H, H-2), 7.91 (s, *J* = 2.2 Hz, 1H, H-6), 6.88 (d, *J* = 8.6 Hz, 1H, H-3), 5.33 (t, *J* = 7.2 Hz, 1H, H-2′), 5.12 (dt, *J* = 17.4, 6.3 Hz, 2H, H-6′, H-10′), 3.91 (s, 3H, H-7), 3.36 (d, *J* = 7.2 Hz, 2H, H-1′), 2.21—1.84 (m, *J* = 16.7, 8.2 Hz 8H, H-4′, H-5′, H-8′, H-9′), 1.73 (s, 3H, H-13′), 1.67 (s, 3H, H-14′), 1.61 (s, 3H, H-15′), 1.60 (s, 3H, H-12′). APT (100 MHz, CDCl_3_): δ_C_ 171.8 (C=O; C-8), 161.7 (C-1), 136.6 (C-3′), 134.9 (C-7′), 131.3 (C-6), 131.0 (C-11′), 130.1 (C-2), 130.0 (C-5), 124.3 (C-6′), 124.0 (C-10′), 121.4 (C-2′), 121.2 (C-4), 109.4 (C-3), 55.4 (C-7), 39.6 (C-4′), 39.5 (C-5′), 28.1 (C-1′), 26.6 (C-8′), 26.5 (C-9′), 25.5 (C-13′), 17.5 (C-14′), 15.9 (C-15′), 15.8 (C-12′). The spectroscopic data were consistent with those reported in the literature for 3-farnesyl-4-methoxybenzoic acid [59]. The spectroscopic data can be consulted in Figures S1 and S2 Supplementary Material.

3-farnesyl-4-hydroxybenzoic acid (**2**): Green solid, melting point (m.p.): 52–54 °C. ^1^H-NMR (400 MHz, CDCl_3_): δ_H_ 7.91 (d, *J* = 3.2 Hz, 1H, H-6), 7.90 (s, 1H, = 9.0, 3.2 Hz, H-2), 6.85 (d, *J* = 9.0 Hz, 1H, H-3), 5.34 (t, *J* = 6.9 Hz, 1H, H-2′), 5.09 (dt, *J* = 5.5, 3.8 Hz, 2H, H-6′, H-10′), 3.42 (d, *J* = 7.1 Hz 2H, H-1′), 2.17–1.95 (m, 8H, *J* = 5.5, 3.8 Hz, H-4′, H-5′, H-8′, H-9′), 1.79 (s, 3H, H-13′), 1.67 (s, 3H, H-14′), 1.60 (s, 6H, H-15′, H-12′). APT (100 MHz, CDCl_3_): δ_C_ 171.8 (C=O, C-8), 159.6 (C-4), 139.6 (C-3′), 135.7 (C-7′), 132.6 (C-6), 131.3 (C-11′), 130.6 (C-2), 126.8 (C-5), 124.4 (C-10′), 123.5 (C-6′), 121.7 (C-1), 120.3 (C-2′), 115.8 (C-3), 39.7 (C-4′), 39.7 (C-8′), 29.7 (C-1′), 26.7 (C-9′), 26.3 (C-5′), 25.7 (C-12′), 17.9 (C-15′), 16.3 (C-14′), 16.0 (C-13′). The spectroscopic data were consistent with those reported in the literature for 3-farnesyl-4-hydroxybenzoic acid [61,63,64,65]. The spectroscopic data can be consulted in Figures S3 and S4 Supplementary Material.

2-farnesylhydroquinone (**3**): White solid, melting point (m.p.): 52–54 °C. ^1^H-NMR (400 MHz, CDCl_3)_: δ_H_ 6.67 (d, *J* = 8.4 Hz, 1H, H-6), 6.61 (d, *J* = 3.2 Hz, 1H, H-3), 6.59 (dd, *J* = 8.4, 3.2 Hz, 1H, H-5), 5.30 (t, *J* = 7.1 Hz, 1H, H-2′), 5.10 (t, *J* = 6.2 Hz, 2H, H-6′, H-10′), 4.85 (s, 1H, OH), 4.67 (s, 1H, OH), 3.30 (d, *J* = 7.1 Hz, 2H, H-1′), 2.23–1.89 (m, *J* = 6.2 Hz, 8H, H-4′, H-5′, H-8′, H-9′), 1.76 (s, 3H, H-13′), 1.68 (s, 3H, H-12′), 1.60 (s, 6H, H-14′, H-15′). APT (100 MHz, CDCl_3_): δ_C_ 149.1 (C-4), 148.0 (C-1), 138.5 (C-3′), 135.4 (C-7′), 131.2 (C-11′), 128.1 (C-2), 124.2 (C-10′), 123.5 (C-6′), 121.1 (C-2′), 116.4 (C-3), 116.4 (C-6), 113.6 (C-5), 39.5 (C-4′)(C-8′), 29.5 (C-1′), 26.5 (C-9′), 26.2 (C-5′), 25.5 (C-12′), 17.5 (C-15′), 16.0 (C-13′), 15.9 (C-14′). The spectroscopic data were consistent with those reported in the literature for 2-farnesylhydroquinone [52]. The spectroscopic data can be consulted in Figures S5 and S6 Supplementary Material.

### 3.4. Strains and Bacterial Growth Conditions

For the long-term conservation of the morphological, physiological, and genetic characteristics of the strains, the freezing method was used at −70 °C and 10% glycerol in Luria Bertani (LB) broth was used as cryoprotectant. The growth temperature for *P. aeruginosa* was 37 °C and for *C. violaceum,* it was 30 °C.

### 3.5. Evaluation of Bacterial Growth on P. aeruginosa

The determination of the bacterial susceptibility of *P. aeruginosa* at extract, fractions, and compounds of *P. bogotense* was performed following the methodology of the Clinical & Laboratory Standards Institute (CLSI) protocol with some modifications [83]. For the extract and fractions, concentrations of 1000, 500 and 250 µg/mL (final in the well), and the isolated compounds 250, 125 and 62.5 µg/mL previously solubilized in dimethyl sulfoxide, DMSO was evaluated. A 96-well plate was used in which 2 µL of each treatment and 2 µL of the inoculum were added to obtain a concentration of 1 × 10^5^ CFU/mL in the final well, which was made up to 200 µL with LB broth. In the assay, five replicates were performed with two independent repetitions. Gentamicin (2 µg/mL), pure DMSO (solvent for the extract), and medium, extract, and growth of *P. aeruginosa* were used as controls. After 24 h, readings were taken at OD 600 nm, and the concentrations that had no inhibitory effect were identified compared to the control.

### 3.6. Biofilm Formation and Quantification Assay in P. aeruginosa

Evaluation of *P. aeruginosa* biofilm formation was performed by the crystal violet staining method [84,85]. A *P. aeruginosa* culture was prepared and left overnight (24 h) at 37 °C at 180 rpm in an orbital shaker incubator. In a 96-well plate, the extract and fractions were added at the same concentrations evaluated in the previous assay that did not show inhibitory effects on bacterial growth were added, and a static incubation was performed for 24 h. Subsequently, the planktonic cells were discarded, and the plate was washed twice with PBS and allowed to dry completely for half an hour. Next, 250 µL of crystal violet (0.1%) was added for 15 min, discarded, and washed three times with PBS to remove excess dye. Finally, 250 µL of ethanol was added to solubilize the crystal adhering to the biofilm. For all assays, five replicates were performed with two independent repetitions. Quercetin (3.9 µg/mL), pure DMSO (solvent of the extract or compound) and medium, extract or compound, and *P. aeruginosa* biofilm formation were used as controls. Readings were taken at an OD of 570 nm, and the percentage of biofilm formation was calculated (Equation (1)).

(1) $$\%\;Biofilm\;formation=\left(\frac{Abs\;treatment}{Abs\;control}\right)\times 100$$

### 3.7. Quorum Sensing Inhibition Assay: Quantification of Violacein Production from C. violaceum

The evaluation of quorum sensing detection in *C. violaceum* was performed by the violacein quantification method described previously [86,87]. A bacterial suspension of *C. violaceum* was prepared in LB broth and adjusted to an OD of 0.4 nm. The extract and fractions were added at the same concentrations evaluated in the biofilm assay. It was left to incubate for 24 h at 30 °C. After this time, the culture was centrifuged at 15,000 rpm for 10 min to separate the cells, and the supernatant was discarded. 500 µL of DMSO was added to solubilize the pigment and centrifuged again at 15,000 rpm for 10 min. A volume of 200 µL of the supernatant was taken, and its absorbance was read at 585 nm in a 96-well plate using a microplate reader. Thymol (100 µg/mL), pure DMSO (solvent of the extract or compound), and medium, compound, and bacteria (*C. violaceum*) were used as controls. Five replicates with two independent repetitions were performed for all assays, and the results were expressed as percent violacein production (Equation (2)).

(2) $$\%\;Violacein\;production=\left(\frac{Abs\;treatment}{Abs\;control}\right)\times 100$$

### 3.8. Assays on Virulence Factors in P. aeruginosa

The evaluation of compounds isolated from *P. bogotense* extract on virulence factors in *P. aeruginosa* was performed following previously described methodologies [88]. An overnight culture of *P. aeruginosa* was placed in contact with different concentrations of the compounds (250, 125, and 62.5 µg/mL). After the 24 h incubation, the cultures were centrifuged at 10,000 rpm for 10 min, and the supernatant was used to quantify the production of proteases, elastases, and the pigment pyocyanin. For all assays, cinnamic acid (250 µg/mL), pure DMSO (compound solvent), and medium, compound, and bacteria (*P. aeruginosa*) were used as controls; and five replicates with two independent repetitions were carried out (Equation (3)).

(3) $$\%\;Production=\left(\frac{Abs\;treatment}{Abs\;control}\right)\times 100$$

#### 3.8.1. Quantification of Pyocyanin Production

The determination of pyocyanin production was performed using the method described previously [88]. The pigment was extracted from 750 µL of the supernatant with 375 µL of chloroform, the supernatant was removed and the organic layer (blue color) was acidified with 300 µL of 0.2 M HCl. The supernatant took a pink coloration, and from this, 150 µL was taken, which was neutralized with 150 µL of a tris buffer at 200 mM to be able to read absorbance at 390 nm.

#### 3.8.2. Quantification of Elastase Production

Protease production was evaluated following the methodology described previously [88]. Twenty-five µL of the supernatant was taken and 225 µL of 100 mM tris buffer pH 7.5, supplemented with 10 mg/mL elastin red Congo (ERC) was added. This mixture was incubated for 3 h at 37 °C under agitation. After this time, PBS buffer at pH 6.0 was added and allowed to cool for 2 min to stop the reaction. Finally, the mixture was centrifuged at 10,000 rpm for 10 min to separate the insoluble ERC and measured absorbance at 495 nm.

#### 3.8.3. Quantification of Protease Production

Proteolytic activity was performed using protease production and quantification by the method previously described [88]. 37.5 µL of the supernatant was taken and a solution of 100 mM tris buffer pH 8.0 supplemented with 0.3% azocasein was added and incubated for one hour at 37 °C statically. Subsequently, trichloroacetic acid (TCA 10%) was added to stop the reaction and insoluble azocasein was centrifuged at 10,000 rpm for 10 min to measure absorbance at 490 nm.

### 3.9. Data Analysis

The one-way ANOVA statistical test was performed considering the assumptions of the test (normality, homogeneity of variances, independence, randomness, and outliers) to determine if there were significant differences in the trials. The data that presented significant differences were subjected to additional multiple comparison tests, such as Duncan for normal data, to confirm in which group the differences occurred. These analyses were performed in the R studio statistical program. All the results reported correspond to the mean of five replicates and their respective standard deviation, using a statistical significance of *p* < 0.05.

## 4. Conclusions

This study represents the first report on the isolation of two benzoic acid derivatives (3-farnesyl-4-methoxybenzoic acid **1** and 3-farnesyl-4-hydroxybenzoic acid **2**) from the aerial part of *P. bogotense*. Furthermore, it is the first time that the antibacterial and antiQS activity of the ethanolic extract of *P. bogotense* and its isolated compounds has been tested against *P. aeruginosa* and *C. violaceum*. Our findings indicate that chemical components of *P. bogotense* have the potential to attenuate the QS system of *P. aeruginosa*, mainly compound **2** (3-farnesyl-4-hydroxybenzoic acid), which exhibits inhibition of violacein, pyocyanin, elastases, protease and biofilm training. Therefore, the phenolic compounds identified from *P. bogotense*, capable of altering the bacterial QS, could be used in combination with conventional antibiotics to increase the efficiency of current antimicrobials by reducing the minimum inhibitory concentration, reducing resistance to antimicrobials and drug side effects.

## Data Availability

Not applicable.

## Supplementary Materials

The following supporting information can be downloaded at: https://www.mdpi.com/article/10.3390/plants12091901/s1, Scheme S1: Isolation scheme of compounds **1** to **3** from *P. bogotense*; Tables S1–S3: supplementary data on the effect of the extract and fractions on susceptibility bioassays; biofilm formation; violacein production, Tables S4 and S5: supplementary data on the effect of the compounds of *P. bogotense* on susceptibility bioassays; biofilm formation; violacein production and virulence factor production; Figures S1–S6: ^1^H-NMR and APT spectra of compounds **1** to **3**.
