# Supplementary material for: Antiquorum and Antibiofilm Activities of Piper bogotense C. DC. against Pseudomonas aeruginosa and Identification of Bioactive Compounds
Source: Plants (Basel). 2023 May 6;12(9):1901. doi: 10.3390/plants12091901 (PMC10180994; doi:10.3390/plants12091901)
Supplement: Supplementary file 1 [file plants-12-01901-s001.zip › plants-2379186-supplementary.pdf]

Supplementary materials

Table of content

1. Phytochemical study carried out on aerial part from *Piper bogotense.*

Scheme S1. Isolation scheme of compounds **1** a **3** from *Piper bogotense*.

2. Percentages of violacein production of *C. violaceum*, growth and biofilm formation of *P. aeruginosa* in the presence of extract and fractions of *P. bogotense*

Table S1. Percent of growth of *P. aeruginosa* on exposure to extract and fractions from *P. bogotense*.

Table S2. Percent of violacein production of *C. violaceum* on exposure to extract and fractions from *P. bogotense*.

Table S3. Percent of biofilm formation in *P. aeruginosa* on exposure to extract and fractions from *P. bogotense*.

3. NMR spectra data from compounds isolated from *Piper bogotense.*

Figure S1. ^{1}H-NMR spectra of 4-methoxy-3-farnesylbenzoic acid (**1**)

Figure S2. APT spectra of 4-methoxy-3-farnesylbenzoic acid (**1**)

Figure S3. ^{1}H-NMR spectra of 4-hydroxy-3-farnesylbenzoic acid (**2**)

Figure S4. APT spectra of 4-hydroxy-3-farnesylbenzoic acid (**2**)

Figure S5. ^{1}H-NMR spectra of 2-farnesylhydroquinone (**3**)

Figure S6. APT spectra of 2-farnesylhydroquinone (**3**)

4. Percentages of violacein production of *C. violaceum*, growth, biofilm formation and production of virulence factors of *P. aeruginosa* in the presence of *P. bogotense* compounds

Table S4. Percent of growth of *P. aeruginosa* on exposure to compounds from *P. bogotense*.

Table S5. Effect of compounds of *P. bogotense* against *C. violaceum* quorum sensing and biofilm formation and production of virulence factors of *P. aeruginosa*

1. Isolation compounds

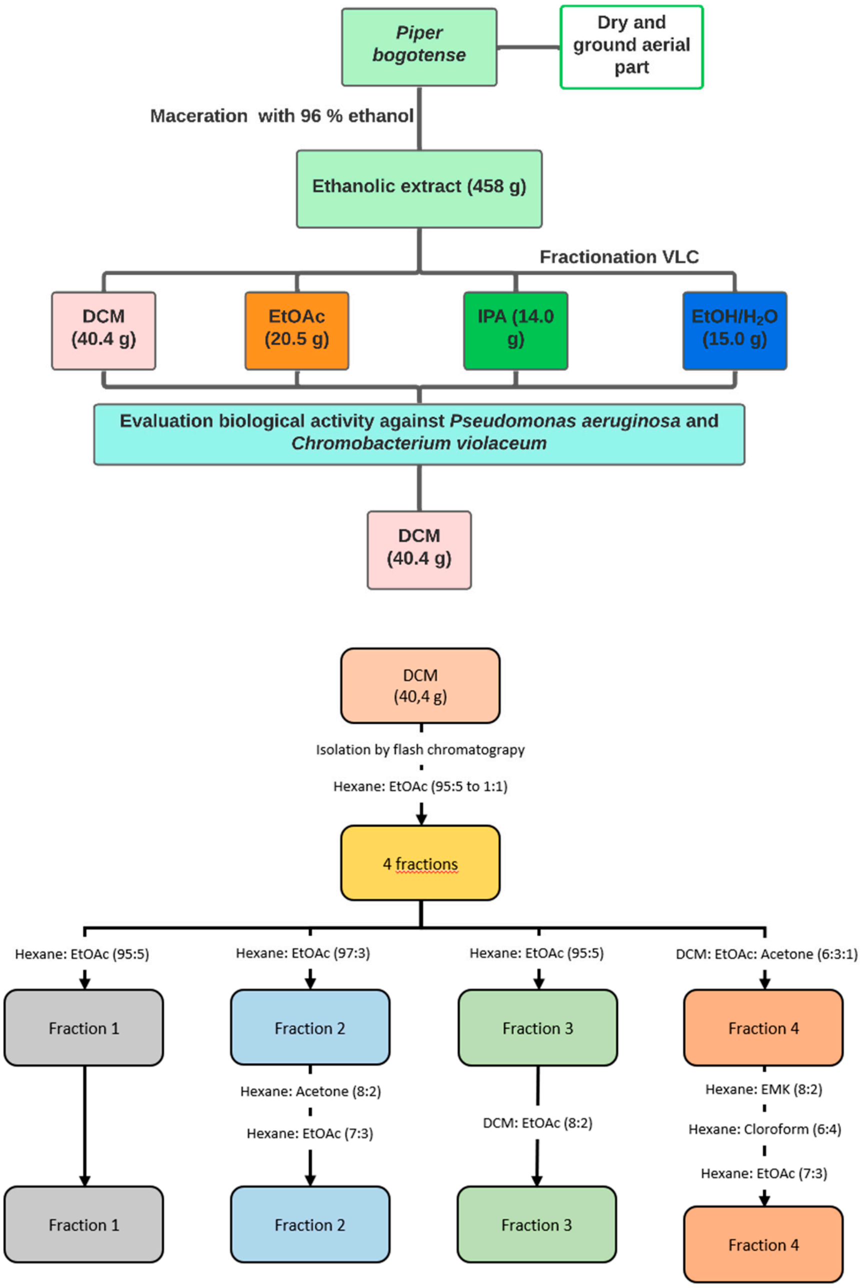

Scheme S1. Isolation scheme of compounds **1** a **3** from *Piper bogotense*.

Table S1. Percent of growth of *P. aeruginosa* on exposure to extract and fractions from *P. bogotense*.

Treatment	Concentrations μg/mL

	1000	500	250
**Extract**	101.9 ± 1.0	98.8 ± 2,5	95.6 ± 2.1
**DCM**	107.5 ± 5.4	96.3 ± 2.3	95.1 ± 0.3
**EtOAc**	102.3 ± 4.2	108.7 ± 5.1	110.8 ± 4.7
**IPA**	106.8 ± 3.1	107.6 ± 2.1	104.3 ± 2.9
**EtOH:H$_2$O**	129.5 ± 2.8	123.6 ± 2.9	118.6 ± 4.9
**Gentamicine 2 µg/mL**	100 ± 0.8		

Table S2. Percent of violacein production of *C. violaceum* on exposure to extract and fractions from *P. bogotense*.

**Treatment**	**Concentrations µg/mL**		
	**1000**	**250**	**62.5**
**Extract**	4.2 ± 1.1*	38.7 ± 2.7*	77.9 ± 4.3*
**DCM**	35.1 ± 6.1*	35.5 ± 2.8*	73.3 ± 6.1*
**EtOAc**	46.1 ± 9.9*	64.9 ± 7.5*	85.9 ± 7.2
**IPA**	33.3 ± 7.8*	0.6 ± 0.5*	0.9 ± 0.7*
**EtOH:H$_2$O**	0.7 ± 0.6*	83.8 ± 9.2	90.4 ± 7.5
**Thymol**	15 ± 0.5		

Data are represented the mean ± standard deviation of five independent replicates. *Indicate a significant difference according to Duncan's test ($p < 0.05$).

Table S3. Percentage of biofilm formation in *P. aeruginosa* on exposure to extract and fractions from *P. bogotense*.

**Treatment**	**Concentrations µg/mL**		
	**1000**	**250**	**62.5**
**Extract**	71.5 ± 14.2*	33.1 ± 0.8*	19.8 ± 1.4*
**DCM**	44.5 ± 15.6*	18.9 ± 11.1*	88.1 ± 9.3
**EtOAc**	95.7 ± 3.6	76.8 ± 9.1*	73.1 ± 8.1*
**IPA**	105.9 ± 9.9	94.1 ± 3.9	101.5 ± 5.9
**EtOH:H$_2$O**	107.1 ± 8.1	98.1 ± 2.6	93.3 ± 5.5
**Quercetin 3.9 µg/mL**	13.7 ± 3.9		

Data are represented the mean ± standard deviation of five independent replicates. *Indicate a significant difference according to Duncan's test ($p < 0.05$).

2. NMR spectra from phytochemistry isolation

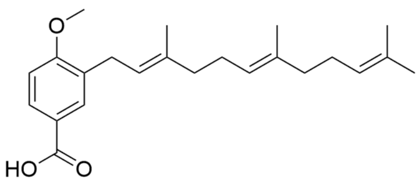

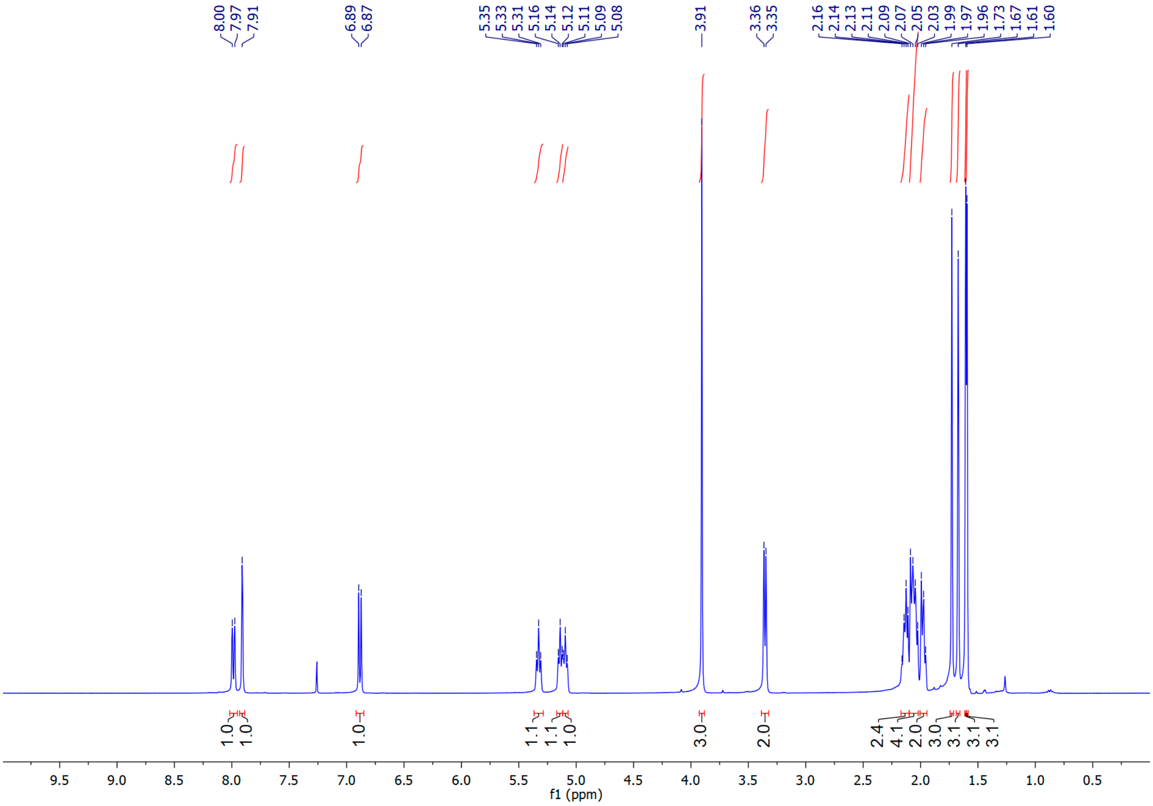

Figure S1. ^{1}H-NMR spectra of 3-farnesyl-4-methoxybenzoic acid (**1**)

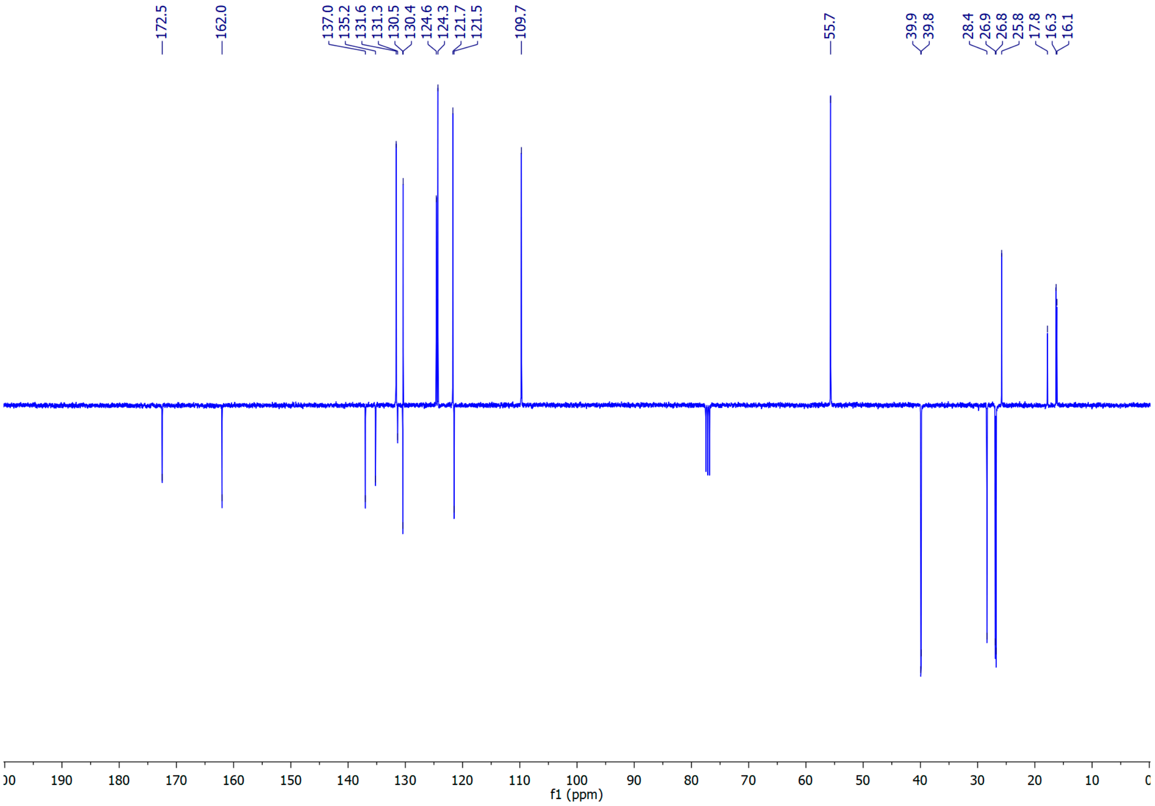

Figure S2. APT spectra of 3-farnesyl-4-methoxybenzoic acid (**1**)

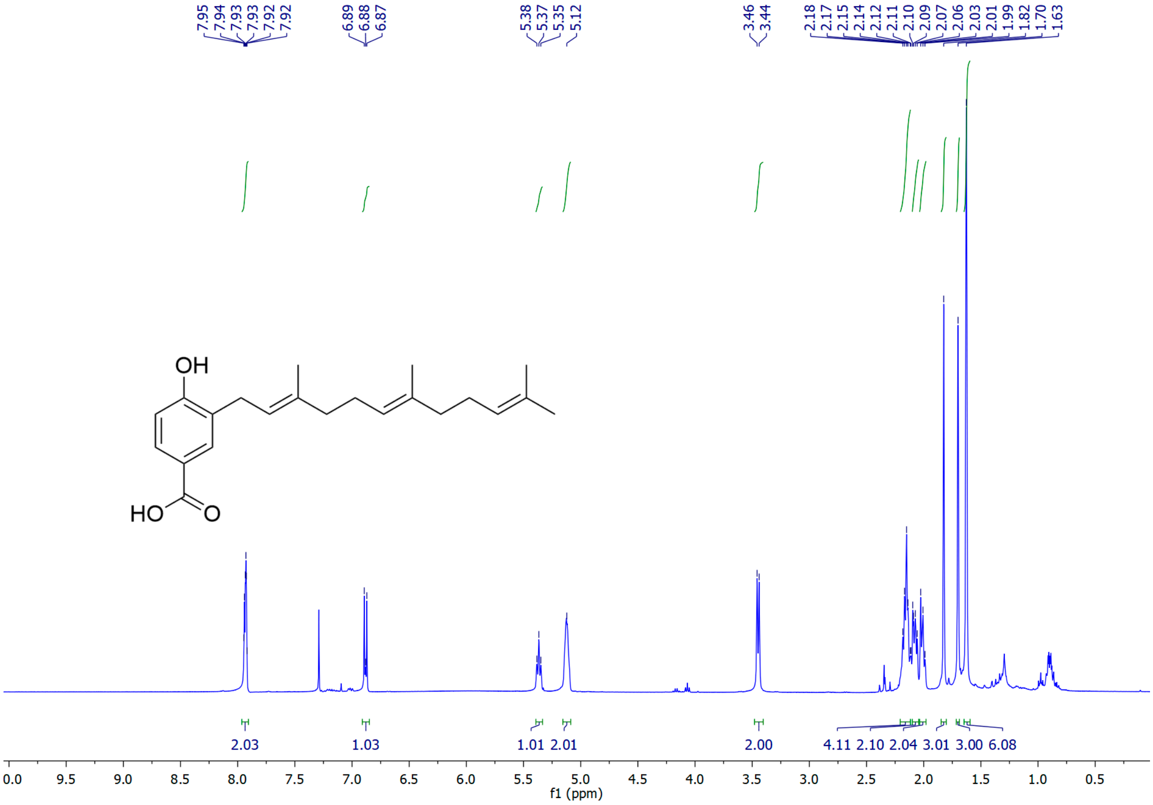

Figure S3. ^{1}H-NMR spectra of 3-farnesyl-4-hydroxybenzoic acid (**2**)

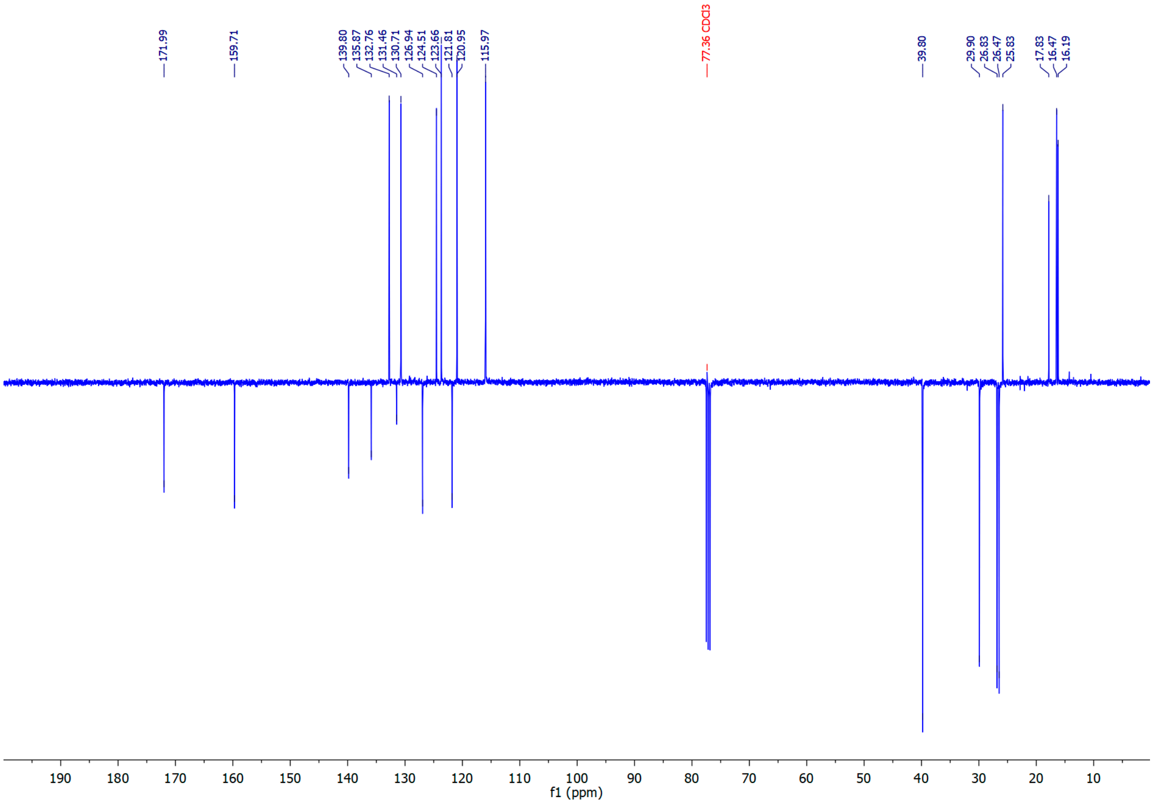

Figure S4. APT spectra of 3-farnesyl-4-hydroxybenzoic acid (**2**)

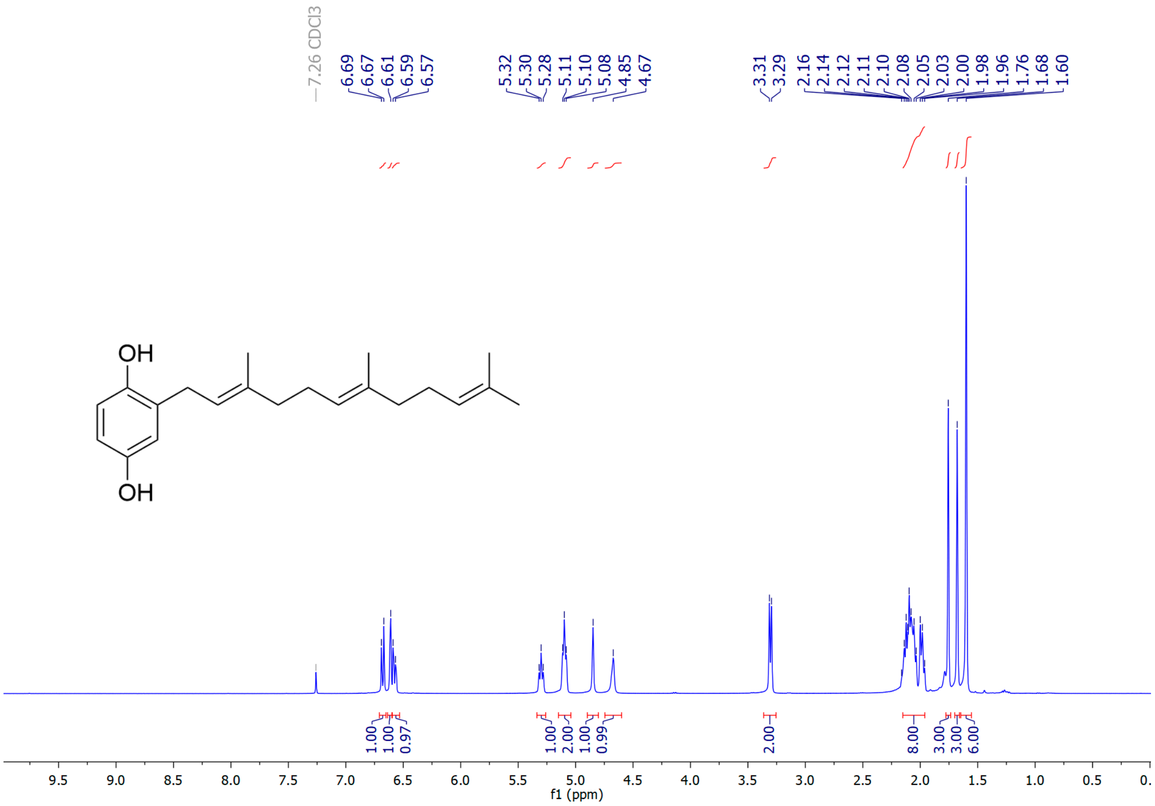

Figure S5. ^{1}H-NMR spectra of 2-farnesylhydroquinone (**3**)

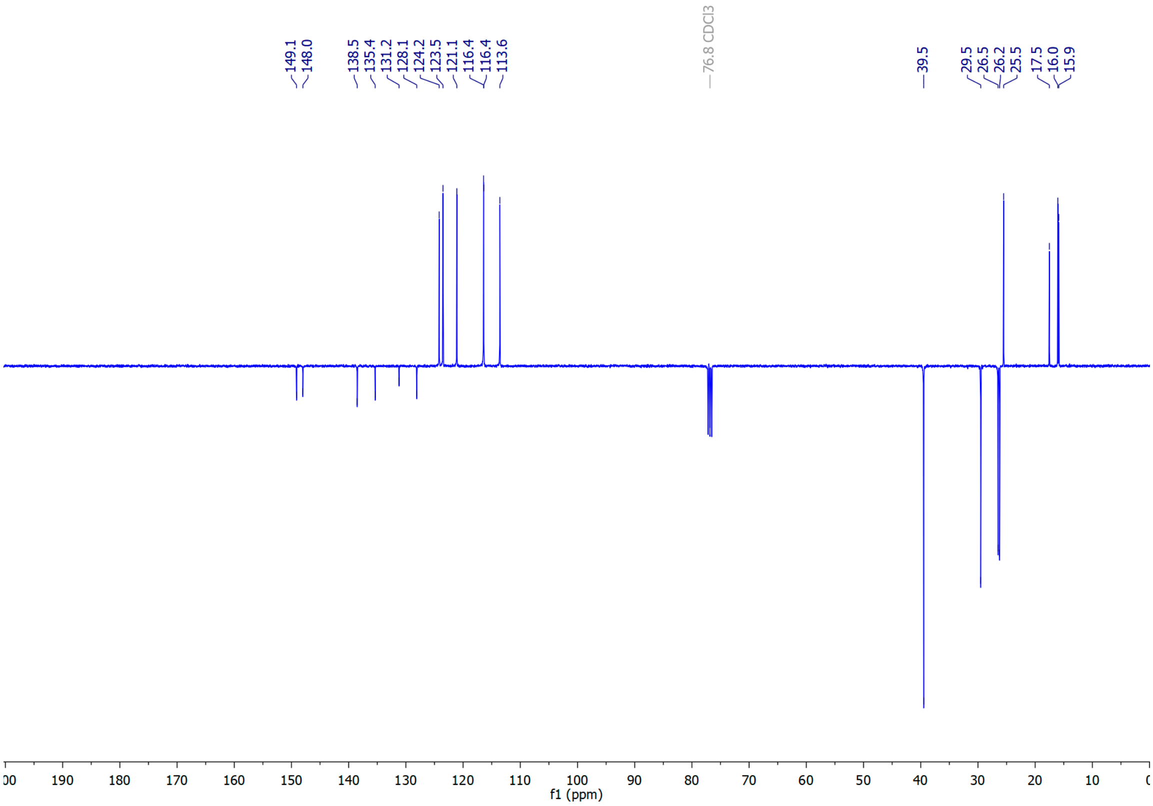

Figure S6. APT spectra of 2-farnesylhydroquinone (**3**)

Table S4. Percent of growth of *P. aeruginosa* on exposure to compounds from *P. bogotense*.

Compound	Concentrations µg/mL		
	**250**	**125**	**62.5**
**1**	108.3 ± 4.1	104.5 ± 4.8	114.7 ± 8.9
**2**	112.9 ± 4.0	102.3 ± 2.0	108.3 ± 4.9
**3**	97.4 ± 2.3	101.5 ± 1.3	102.7 ± 2.2
**Gentamicine**	5.2 ± 0.8		

Table S5. Effect of compounds of *P. bogotense* against *C. violaceum* quorum sensing and biofilm formation and production of virulence factors of *P. aeruginosa.*

Compound	Bioassay	Concentrations µg/mL		
		250	125	62.5
1	Violacein	75.4 ± 3.8	75.9 ± 3.5	89.5 ± 3.5
	Biofilm	207.5 ± 10.3*	155.1 ± 18.8*	112.5 ± 28.6
	Piocianina	78.3 ± 17.2	81.7 ± 2.3	81.4 ± 15.3
	Proteasas	91.1 ± 1.1	92.7 ± 4.4	93.3 ± 2.1
	Elastasas	87.8 ± 22.3	99.0 ± 13.3	95.4 ± 18.2
2	Violacein	43.8 ± 9.1	49.7 ± 4.4	83.8 ± 4.8
	Biofilm	82.3 ± 29.9	178.3 ± 24.2*	157.1 ± 13.4*
	Piocianina	54.0 ± 20.0*	58.8 ± 18.7*	39.7 ± 7.8*
	Proteasas	80.6 ± 16.4	79.6 ± 19.2	87.8 ± 16.3
	Elastasas	60.2 ± 16.4*	88.1 ± 13.5	101.6 ± 17.5
3	Violacein	68.3 ± 6.1	70.2 ± 10.1	79.1 ± 16.1
	Biofilm	142.8 ± 20.8*	115.8 ± 4.2	73.3 ± 11.0
	Piocianina	33.2 ± 20.0*	69.6 ± 21.3	78.9 ± 2.5
	Proteasas	73.9 ± 13.9*	84.3 ± 11.0	101.2 ± 4.3
	Elastasas	51.4 ± 18.3*	100.8 ± 20.8	93.9 ± 22.4

Data are represented the mean ± standard deviation of five independent replicates. *Indicate a significant difference according to Duncan's test ($p < 0.05$).
